# Supplementary material for: Investigation of the Genomic and Pathogenic Features of the Potentially Zoonotic Streptococcus parasuis
Source: Pathogens. 2021 Jul 2;10(7):834. doi: 10.3390/pathogens10070834 (PMC8308872; doi:10.3390/pathogens10070834)
Supplement: Supplementary file 1 [file pathogens-10-00834-s001.zip › supple 6.28/Supplemental Material table S1.pdf]

Table S1. Sequence similarity of 16S rRNA gene and housekeeping genes and ANI values among genomes that the present study used.

| | | Sequence similarity (%) of 16S rRNA gene with strains No. | | | | | | | | | | | | | | | | | |
|---|---|---|---|---|---|---|---|---|---|---|---|---|---|---|---|---|---|---|---|
| No. | Strains | 1 | 2 | 3 | 4 | 5 | 6 | 7 | 8 | 9 | 10 | 11 | 12 | 13 | 14 | 15 | 16 | 17 | 18 |
| 1 | *S. parasuis* BS26 | | | | | | | | | | | | | | | | | | |
| 2 | *S. parasuis* BS27 | 100.0 | | | | | | | | | | | | | | | | | |
| 3 | *S. parasuis* SUT-7 | 99.4 | 99.4 | | | | | | | | | | | | | | | | |
| 4 | *S. parasuis* SUT-286 | 98.9 | 98.9 | 99.2 | | | | | | | | | | | | | | | |
| 5 | *S. parasuis* SUT-328 | 99.5 | 99.5 | 99.2 | 99.2 | | | | | | | | | | | | | | |
| 6 | *S. parasuis* 86-5192 | 98.9 | 98.9 | 100.0 | 100.0 | 99.2 | | | | | | | | | | | | | |
| 7 | *S. parasuis* 88-1861 | 99.2 | 99.2 | 99.2 | 99.2 | 99.6 | 99.1 | | | | | | | | | | | | |
| 8 | *S. parasuis* 89-4109-1 | 98.9 | 98.9 | 100.0 | 100.0 | 99.2 | 100.0 | 99.5 | | | | | | | | | | | |
| 9 | *S. parasuis* 4253 | 99.3 | 99.3 | 99.5 | 99.5 | 99.0 | 99.5 | 98.8 | 99.5 | | | | | | | | | | |

| | | | | | | | | | | | | | | | | | | |
|---|---|---|---|---|---|---|---|---|---|---|---|---|---|---|---|---|---|---|
| 10 | *S. parasuis* 2674 | 99.2 | 99.2 | 99.2 | 99.2 | 99.6 | 99.1 | 100.0 | 99.1 | 99.1 | | | | | | | | |
| 11 | *S. parasuis* 10-36905 | 98.9 | 98.9 | 100.0 | 100.0 | 99.2 | 100.0 | 99.5 | 99.5 | 99.5 | 99.6 | | | | | | | |
| 12 | *S. parasuis* SUT-447 | 99.6 | 99.6 | 98.8 | 98.8 | 99.3 | 98.8 | 99.1 | 98.8 | 98.9 | 99.1 | 98.8 | | | | | | |
| 13 | *S. parasuis* SUT-462 | 98.9 | 98.9 | 100.0 | 100.0 | 99.2 | 100.0 | 99.1 | 100.0 | 99.5 | 99.1 | 100.0 | 98.8 | | | | | |
| 14 | *S. parasuis* SUT-479 | 99.4 | 99.4 | 99.3 | 99.3 | 99.9 | 99.3 | 99.7 | 99.3 | 98.9 | 99.7 | 99.3 | 99.4 | 99.3 | | | | |
| 15 | *S. parasuis* SUT-481 | 98.9 | 98.9 | 99.9 | 99.9 | 99.2 | 99.9 | 99.1 | 99.9 | 99.5 | 99.1 | 99.9 | 98.8 | 99.9 | 99.4 | | | |
| 16 | *S. parasuis* SUT-483 | 99.1 | 99.1 | 99.6 | 99.6 | 99.6 | 99.6 | 99.2 | 99.6 | 99.1 | 99.2 | 99.6 | 99.0 | 99.6 | 99.5 | 99.5 | | |
| 17 | *S. suis* NCTC10234 | 96.6 | 96.6 | 97.6 | 97.6 | 97.0 | 97.6 | 97.1 | 97.1 | 97.1 | 97.3 | 97.6 | 96.5 | 97.6 | 97.0 | 97.6 | 97.3 | |
| 18 | *E. faecalis* JCM_5803 | 89.1 | 89.1 | 89.1 | 89.1 | 88.9 | 89.4 | 89.3 | 89.3 | 89.3 | 89.3 | 89.4 | 88.9 | 89.4 | 89.3 | 89.5 | 89.3 | 89.1 |

| | | Sequence similarity (%) of ***groEL*** with strains No. | | | | | | | | | | | | | |
|---|---|---|---|---|---|---|---|---|---|---|---|---|---|---|---|
| No. | Strains | 1 | 2 | 3 | 4 | 5 | 6 | 7 | 8 | 9 | 10 | 11 | 12 | 13 | 14 |
| 1 | *S. parasuis* BS26 | | | | | | | | | | | | | | |
| 2 | *S. parasuis* BS27 | 100.0 | | | | | | | | | | | | | |
| 3 | *S. parasuis* SUT-7 | 96.9 | 96.9 | | | | | | | | | | | | |
| 4 | *S. parasuis* SUT-286 | 97.6 | 97.6 | 97.8 | | | | | | | | | | | |
| 5 | *S. parasuis* SUT-319 | 94.2 | 94.2 | 94.4 | 95.0 | | | | | | | | | | |
| 6 | *S. parasuis* SUT-328 | 94.2 | 94.2 | 94.4 | 95.0 | 100.0 | | | | | | | | | |
| 7 | *S. parasuis* SUT-380 | 94.2 | 94.2 | 94.4 | 95.0 | 100.0 | 100.0 | | | | | | | | |
| 8 | *S. parasuis* 86-5192 | 96.0 | 96.0 | 96.0 | 96.9 | 94.0 | 94.0 | 94.0 | | | | | | | |
| 9 | *S. parasuis* 88-1861 | 96.1 | 96.1 | 96.2 | 96.9 | 94.2 | 94.2 | 94.2 | 99.5 | | | | | | |
| 10 | *S. parasuis* 89-4109-1 | 96.0 | 96.0 | 96.0 | 96.9 | 94.0 | 94.0 | 94.0 | 100.0 | 99.5 | | | | | |

| | | | | | | | | | | | | | | | |
|---|---|---|---|---|---|---|---|---|---|---|---|---|---|---|---|
| 11 | *S. parasuis* 4253 | 98.4 | 98.4 | 97.2 | 97.8 | 94.4 | 94.4 | 94.4 | 96.2 | 96.5 | 96.2 | | | | |
| 12 | *S. parasuis* 2674 | 94.2 | 94.2 | 94.6 | 95.1 | 99.7 | 99.7 | 99.7 | 94.0 | 94.3 | 94.0 | 94.5 | | | |
| 13 | *S. parasuis* 10-36905 | 96.0 | 96.0 | 96.0 | 96.9 | 94.0 | 94.0 | 94.0 | 100.0 | 99.5 | 100.0 | 96.2 | 94.0 | | |
| 14 | *S. suis* NCTC10234 | 91.5 | 91.5 | 92.0 | 91.6 | 93.0 | 93.0 | 93.0 | 91.9 | 91.7 | 91.9 | 91.4 | 93.0 | 91.9 | |

| | | **Sequence similarity (%) of *gryB* with strains No.** | | | | | | | | | | | | | |
|---|---|---|---|---|---|---|---|---|---|---|---|---|---|---|---|
| No. | Strains | 1 | 2 | 3 | 4 | 5 | 6 | 7 | 8 | 9 | 10 | 11 | 12 | 13 | 14 |
| 1 | *S. parasuis* BS26 | | | | | | | | | | | | | | |
| 2 | *S. parasuis* BS27 | 100.0 | | | | | | | | | | | | | |
| 3 | *S. parasuis* SUT-7 | 97.5 | 97.5 | | | | | | | | | | | | |
| 4 | *S. parasuis* SUT-286 | 97.6 | 97.6 | 97.3 | | | | | | | | | | | |
| 5 | *S. parasuis* SUT-319 | 97.6 | 97.6 | 98.4 | 97.6 | | | | | | | | | | |
| 6 | *S. parasuis* SUT-328 | 97.6 | 97.6 | 98.4 | 97.6 | 100.0 | | | | | | | | | |

| | | | | | | | | | | | | | | |
|---|---|---|---|---|---|---|---|---|---|---|---|---|---|---|
| 7 | *S. parasuis* SUT-380 | 98.0 | 98.0 | 97.9 | 98.1 | 98.6 | 98.6 | | | | | | | |
| 8 | *S. parasuis* 86-5192 | 97.8 | 97.8 | 97.7 | 97.8 | 98.2 | 98.2 | 99.6 | | | | | | |
| 9 | *S. parasuis* 88-1861 | 97.5 | 97.5 | 97.9 | 98.1 | 98.0 | 98.0 | 98.3 | 97.8 | | | | | |
| 10 | *S. parasuis* 89-4109-1 | 97.5 | 97.5 | 98.3 | 97.3 | 99.1 | 99.1 | 99.0 | 98.6 | 98.1 | | | | |
| 11 | *S. parasuis* 4253 | 99.1 | 99.1 | 97.2 | 97.4 | 97.2 | 97.2 | 97.8 | 97.7 | 97.2 | 97.4 | | | |
| 12 | *S. parasuis* 2674 | 97.3 | 97.3 | 97.4 | 98.9 | 97.7 | 97.7 | 98.1 | 97.8 | 98.5 | 97.6 | 97.0 | | |
| 13 | *S. parasuis* 10-36905 | 97.5 | 97.5 | 97.2 | 99.4 | 97.5 | 97.5 | 98.2 | 98.0 | 98.2 | 97.5 | 97.2 | 97.2 | |
| 14 | *S. suis* NCTC10234 | 83.9 | 83.9 | 84.1 | 84.1 | 84.1 | 84.1 | 84.3 | 84.3 | 84.1 | 84.2 | 83.7 | 83.7 | 84.1 |

| No. | Strains | **Sequence similarity (%) of *sodA* with strains No.** | | | | | | | | | | | | | |
|---|---|---|---|---|---|---|---|---|---|---|---|---|---|---|---|
| | | 1 | 2 | 3 | 4 | 5 | 6 | 7 | 8 | 9 | 10 | 11 | 12 | 13 | 14 |
| 1 | *S. parasuis* BS26 | | | | | | | | | | | | | | |

| | | | | | | | | | | | | | | |
|---|---|---|---|---|---|---|---|---|---|---|---|---|---|---|
| 2 | *S. parasuis* BS27 | 100.0 | | | | | | | | | | | | |
| 3 | S. parasuis SUT-7 | 96.9 | 96.9 | | | | | | | | | | | |
| 4 | *S. parasuis* SUT-286 | 98.7 | 98.7 | 97.5 | | | | | | | | | | |
| 5 | *S. parasuis* SUT-319 | 98.5 | 98.5 | 97.4 | 98.8 | | | | | | | | | |
| 6 | *S. parasuis* SUT-328 | 98.5 | 98.5 | 97.4 | 98.8 | 100.0 | | | | | | | | |
| 7 | *S. parasuis* SUT-380 | 98.2 | 98.2 | 97.4 | 98.8 | 99.0 | 99.0 | | | | | | | |
| 8 | *S. parasuis* 86-5192 | 97.5 | 97.5 | 98.7 | 98.5 | 97.7 | 97.7 | 100.0 | | | | | | |
| 9 | *S. parasuis* 88-1861 | 96.9 | 96.9 | 99.7 | 97.5 | 97.4 | 97.4 | 98.7 | 98.7 | | | | | |
| 10 | *S. parasuis* 89-4109-1 | 96.7 | 96.7 | 99.8 | 97.4 | 97.2 | 97.2 | 97.2 | 98.5 | 99.5 | | | | |
| 11 | *S. parasuis* 4253 | 99.3 | 99.3 | 96.9 | 98.7 | 98.5 | 98.5 | 98.5 | 97.5 | 96.9 | 96.7 | | | |
| 12 | *S. parasuis* 2647 | 97.9 | 97.9 | 98.4 | 98.5 | 98.4 | 98.4 | 98.4 | 97.7 | 97.5 | 97.9 | 97.9 | | |
| 13 | *S. parasuis* 10-36905 | 96.9 | 96.9 | 100.0 | 97.5 | 97.4 | 97.4 | 97.4 | 98.7 | 99.7 | 99.8 | 96.9 | 97.7 | |
| 14 | *S. suis* NCTC10234 | 83.0 | 83.0 | 84.7 | 84.0 | 84.0 | 84.0 | 84.0 | 84.3 | 84.3 | 84.5 | 83.0 | 84.0 | 84.7 |

| No. | Strains | **Sequence similarity (%) of *recN* with strains No.** | | | | | | | | | | | | | | | | | | | | | | | | | |
|---|---|---|---|---|---|---|---|---|---|---|---|---|---|---|---|---|---|---|---|---|---|---|---|---|---|---|---|
| | | 1 | 2 | 3 | 4 | 5 | 6 | 7 | 8 | 9 | 10 | 11 | 12 | 13 | 14 | 15 | 16 | 17 | 18 | 19 | 20 | 21 | 22 | 23 | 24 | 25 | 26 |
| 1 | *S. parasuis* BS26 | | | | | | | | | | | | | | | | | | | | | | | | | | |
| 2 | *S. parasuis* BS27 | 100.0 | | | | | | | | | | | | | | | | | | | | | | | | | |
| 3 | *S. parasuis* SUT-7 | 98.3 | 98.3 | | | | | | | | | | | | | | | | | | | | | | | | |
| 4 | *S. parasuis* SUT-286 | 97.4 | 97.4 | 97.4 | | | | | | | | | | | | | | | | | | | | | | | |
| 5 | *S. parasuis* SUT-319 | 98.4 | 98.4 | 99.6 | 97.5 | | | | | | | | | | | | | | | | | | | | | | |
| 6 | *S. parasuis* SUT-328 | 98.4 | 98.4 | 99.6 | 97.5 | 100.0 | | | | | | | | | | | | | | | | | | | | | |
| 7 | *S. parasuis* SUT-380 | 98.4 | 98.4 | 99.6 | 97.5 | 100.0 | 100.0 | | | | | | | | | | | | | | | | | | | | |
| 8 | *S. parasuis* 86-5192 | 97.3 | 97.3 | 97.3 | 99.6 | 97.4 | 97.4 | 97.4 | | | | | | | | | | | | | | | | | | | |
| 9 | *S. parasuis* 88-1861 | 97.5 | 97.5 | 97.8 | 98.9 | 97.8 | 97.8 | 97.8 | 98.7 | | | | | | | | | | | | | | | | | | |
| 10 | *S. parasuis* 89-4109-1 | 97.3 | 97.3 | 97.3 | 99.2 | 97.4 | 97.4 | 97.4 | 99.2 | 98.9 | | | | | | | | | | | | | | | | | |
| 11 | *S. parasuis* 4253 | 99.0 | 99.0 | 98.4 | 97.7 | 98.5 | 98.5 | 98.5 | 97.6 | 97.8 | 97.6 | | | | | | | | | | | | | | | | |
| 12 | *S. parasuis* 2674 | 97.6 | 97.6 | 98.3 | 97.4 | 98.3 | 98.3 | 98.3 | 97.3 | 97.4 | 97.1 | 97.8 | | | | | | | | | | | | | | | |
| 13 | *S. parasuis* 10-36905 | 97.3 | 97.3 | 97.3 | 99.6 | 97.4 | 97.4 | 97.4 | 100.0 | 98.7 | 99.2 | 97.6 | 97.3 | | | | | | | | | | | | | | |

| | | | | | | | | | | | | | | | | | | | | | | | | | | |
|---|---|---|---|---|---|---|---|---|---|---|---|---|---|---|---|---|---|---|---|---|---|---|---|---|---|---|
| 14 | *S. parasuis* SUT-443 | 98.1 | 98.1 | 97.7 | 99.7 | 97.8 | 97.8 | 97.8 | 99.5 | 98.7 | 99.1 | 98.5 | 97.9 | 99.5 | | | | | | | | | | | | |
| 15 | *S. parasuis* SUT-447 | 98.7 | 98.7 | 97.9 | 98.0 | 98.0 | 98.0 | 98.0 | 97.8 | 97.9 | 97.9 | 99.1 | 97.9 | 97.8 | 98.1 | | | | | | | | | | | |
| 16 | *S. parasuis* SUT-458 | 97.8 | 97.8 | 97.6 | 99.2 | 97.7 | 97.7 | 97.7 | 99.4 | 98.6 | 99.7 | 98.2 | 97.6 | 99.4 | 99.1 | 97.9 | | | | | | | | | | |
| 17 | *S. parasuis* SUT-462 | 97.8 | 97.8 | 97.6 | 99.2 | 97.7 | 97.7 | 97.7 | 99.4 | 98.6 | 99.8 | 98.2 | 97.6 | 99.4 | 99.1 | 97.9 | 99.9 | | | | | | | | | |
| 18 | *S. parasuis* SUT-479 | 98.0 | 98.0 | 98.6 | 97.9 | 98.7 | 98.7 | 98.7 | 97.7 | 98.2 | 97.7 | 98.4 | 99.0 | 97.7 | 97.8 | 98.0 | 97.7 | 97.7 | | | | | | | | |
| 19 | *S. parasuis* SUT-483 | 98.2 | 98.2 | 99.5 | 97.9 | 100.0 | 100.0 | 100.0 | 97.7 | 98.2 | 97.7 | 98.4 | 98.4 | 97.7 | 97.8 | 98.0 | 97.7 | 97.7 | 98.7 | | | | | | | |
| 20 | *S. parasuis* SUT-488 | 98.0 | 98.0 | 98.8 | 98.3 | 99.1 | 99.1 | 99.1 | 98.1 | 98.4 | 98.1 | 98.4 | 98.2 | 98.1 | 98.2 | 98.0 | 98.1 | 98.1 | 98.3 | 99.1 | | | | | | |
| 21 | *S. parasuis* SUT-503 | 98.0 | 98.0 | 97.8 | 100.0 | 97.9 | 97.9 | 97.9 | 99.6 | 98.8 | 99.2 | 98.4 | 98.0 | 99.6 | 99.7 | 98.0 | 99.2 | 99.2 | 97.9 | 97.9 | 98.3 | | | | | |
| 22 | *S. parasuis* SUT-507 | 97.8 | 97.8 | 97.6 | 99.2 | 97.7 | 97.7 | 97.7 | 99.4 | 98.6 | 99.2 | 98.2 | 97.6 | 99.4 | 99.1 | 97.9 | 99.9 | 100.0 | 97.7 | 97.7 | 98.1 | 99.2 | | | | |
| 23 | *S. parasuis* SUT-516 | 98.2 | 98.2 | 99.5 | 97.9 | 100.0 | 100.0 | 100.0 | 97.7 | 98.2 | 97.7 | 98.4 | 98.4 | 97.7 | 97.8 | 98.0 | 97.7 | 97.7 | 98.7 | 100.0 | 99.1 | 97.9 | 97.7 | | | |
| 24 | *S. parasuis* SUT-523 | 98.1 | 98.1 | 99.4 | 97.8 | 99.9 | 99.9 | 99.9 | 97.6 | 98.1 | 97.6 | 98.3 | 98.3 | 97.6 | 97.7 | 97.9 | 97.6 | 97.6 | 98.6 | 99.9 | 99.0 | 97.8 | 97.6 | 99.9 | | |
| 25 | *S. parasuis* SUT-529 | 97.8 | 97.8 | 97.6 | 99.2 | 97.7 | 97.7 | 97.7 | 99.4 | 98.6 | 99.8 | 98.2 | 97.6 | 99.4 | 99.1 | 97.9 | 99.9 | 100.0 | 97.7 | 97.7 | 98.1 | 99.2 | 100.0 | 97.7 | 97.6 | |
| 26 | *S. suis* NCTC10234 | 77.0 | 77.0 | 77.0 | 77.0 | 77.0 | 77.0 | 77.0 | 77.0 | 77.0 | 76.0 | 77.0 | 77.0 | 77.0 | 75.0 | 75.0 | 75.0 | 75.0 | 75.0 | 75.0 | 75.0 | 75.0 | 75.0 | 75.0 | 75.0 | 75.0 |

| | | ANI values with strains No. | | | | | | | | | | | | | |
|---|---|---|---|---|---|---|---|---|---|---|---|---|---|---|---|
| No. | Strains | 1 | 2 | 3 | 4 | 5 | 6 | 7 | 8 | 9 | 10 | 11 | 12 | 13 | 14 |
| 1 | *S.parasuis* BS26 | | | | | | | | | | | | | | |
| 2 | *S.parasuis* BS27 | 99.98 | | | | | | | | | | | | | |
| 3 | *S.parasuis* SUT-7 | 93.98 | 94.13 | | | | | | | | | | | | |
| 4 | *S.parasuis* SUT-286 | 95.1 | 95.2 | 94.96 | | | | | | | | | | | |
| 5 | *S.parasuis* SUT-319 | 95.3 | 95.23 | 95.15 | 96.44 | | | | | | | | | | |
| 6 | *S.parasuis* SUT-328 | 95.31 | 95.33 | 95.14 | 96.58 | 99.99 | | | | | | | | | |
| 7 | *S.parasuis* SUT-380 | 95.26 | 95.28 | 94.97 | 96.65 | 99.45 | 99.44 | | | | | | | | |
| 8 | *S.parasuis* 86-5192 | 95.14 | 95.04 | 94.3 | 97 | 96.06 | 96.05 | 96.22 | | | | | | | |
| 9 | *S.parasuis* 88-1861 | 93.86 | 93.8 | 94.7 | 95.32 | 94.87 | 94.78 | 94.63 | 95.09 | | | | | | |
| 10 | *S.parasuis* 89-4109-1 | 95.28 | 95.19 | 94.52 | 97.2 | 96.02 | 96.04 | 95.88 | 97.25 | 95.17 | | | | | |
| 11 | *S.parasuis* 4253 | 97.79 | 97.77 | 94.06 | 94.86 | 95.14 | 95.21 | 95.13 | 94.93 | 93.45 | 94.92 | | | | |
| 12 | *S.parasuis* 2674 | 94.02 | 94.02 | 95.13 | 95.63 | 95 | 95 | 94.99 | 95.93 | 95.35 | 95.4 | 93.92 | | | |

| | | | | | | | | | | | | | | |
|---|---|---|---|---|---|---|---|---|---|---|---|---|---|---|
| 13 | *S.parasuis* 10-36905 | 95 | 95 | 94.37 | 97.2 | 95.67 | 95.74 | 95.75 | 97.42 | 95.25 | 97.48 | 94.62 | 95.75 | |
| 14 | *S.suis* NCTC10234 | 83.42 | 83.33 | 84.72 | 84.57 | 84.5 | 84.5 | 84.75 | 84.32 | 84.5 | 84.11 | 84.36 | 84.58 | 84.48 |
