# Supplementary material for: Investigation of the Genomic and Pathogenic Features of the Potentially Zoonotic Streptococcus parasuis
Source: Pathogens. 2021 Jul 2;10(7):834. doi: 10.3390/pathogens10070834 (PMC8308872; doi:10.3390/pathogens10070834)
Supplement: Supplementary file 1 [file pathogens-10-00834-s001.zip › supple 6.28/Supplemental Material table S2.pdf]

Table S2. Survival table of each infected group and statistical data in survival assay.

Survival table

	Id	Time	Status (0: dead; 1: survival)	Cumulative proportion of survival at this time		Cumulative events	Remaining cases
				Estimate	Standard error		
BS26 1	21	8	0	.095	.049	1	19
2	22	10	0	.	.	2	18
3	23	10	0	.850	.080	3	17
4	24	12	0	.800	.089	4	16
5	25	18	0	.	.	5	15
6	26	18	0	.	.	6	14
7	27	18	0	.	.	7	13
8	28	18	0		.	8	12
9	29	18	0	.	.	9	11
10	30	18	0	.	.	10	10
11	31	18	0	.	.	11	9
12	32	18	0	.	.	12	8
13	33	18	0	.	.	13	7
14	34	18	0	.	.	14	6
15	35	18	0	.250	.097	15	5
16	36	24	0	.200	.089	16	4
17	37	36	0	.150	.080	17	3
18	38	72	1	.	.	17	2
19	39	72	1	.	.	17	1
20	40	72	1	.	.	17	0
BS27 1	41	8	0	.900	.067	1	19
2	42	10	0	.850	.080	2	18
3	43	12	0	.	.	3	17
4	44	12	0	.750	.097	4	16
5	45	18	0	.	.	5	15
6	46	18	0	.	.	6	14
7	47	18	0	.	.	7	13
8	48	18	0	.	.	8	12
9	49	18	0	.	.	9	11
10	50	18	0	.	.	10	10
11	51	18	0	.	.	11	9
12	52	18	0	.	.	12	8
13	53	18	0	.	.	13	7
14	54	18	0	.300	.102	14	6
15	55	24	0	.250	.097	15	5
16	56	72	1	.	.	15	4
17	57	72	1	.	.	15	3

Survival table

	Id	Time	Status (0: dead; 1: survival)	Cumulative proportion of survival at this time		Cumulative events	Remaining cases
				Estimate	Standard error		
18	58	72	1	.	.	15	2
19	59	72	1	.	.	15	1
20	60	72	1	.	.	15	0
P1-7 1	1	4	0	.950	.049	1	19
2	2	6	0	.	.	2	18
3	3	6	0	.	.	3	17
4	4	6	0	.	.	4	16
5	5	6	0	.	.	5	15
6	6	6	0	.	.	6	14
7	7	6	0	.	.	7	13
8	8	6	0	.	.	8	12
9	9	6	0	.	.	9	11
10	10	6	0	.	.	10	10
11	11	6	0	.	.	11	9
12	12	6	0	.400	.110	12	8
13	13	8	0	.	.	13	7
14	14	8	0	.	.	4	6
15	15	8	0	.	.	15	5
16	16	8	0	.200	.089	16	4
17	17	18	0	.150	.080	17	3
18	18	72	1	.	.	17	2
19	19	72	1	.	.	17	1
20	20	72	1	.	.	17	0

Mean and median of survival table

	average				median			
			95% confidence interval				95% confidence interval	
	Estimate	Standard error	Lower limit	Upper limit	Estimate	Standard error	Lower limit	Upper limit
BS26	25.700	4.524	16.833	34.567	18.000	1.056	15.930	20.070
BS27	29.700	5.536	18.850	40.550	18.000	1.366	15.322	20.678
P1-7	16.800	5.220	6.569	27.031	6.000	.398	5.219	6.781
total	24.067	3.032	18.125	30.009	18.000	.936	16.165	19.835

a. If the estimate has been censored, it will be limited to the longest survival time.

Overall comparison

		BS26		BS27		P1-7	
		χ^2	Sig.	χ^2	Sig.	χ^2	Sig.
Log Rank (Mantel-Cox)	BS26			.123.	.726	7.334	.007
	BS27	.123	.726			8.005	.005
	P1-7	7.334	.007	8.005	.005		
